# Nano-Biomechanical Study of Spatio-Temporal Cytoskeleton Rearrangements that Determine Subcellular Mechanical Properties and Endothelial Permeability

**DOI:** 10.1038/srep11097

**Published:** 2015-06-18

**Authors:** Xin Wang, Reiner Bleher, Mary E. Brown, Joe G. N. Garcia, Steven M. Dudek, Gajendra S. Shekhawat, Vinayak P. Dravid

**Affiliations:** 1 Department of Materials Science and Engineering, Northwestern University, Evanston, IL, USA. 60208; 2 Department of Medicine, University of Illinois, Chicago, IL, USA. 60612; 3 Arizona Health Sciences Center, The University of Arizona, Tucson, AZ, USA. 210202

## Abstract

The endothelial cell (EC) lining of the pulmonary vascular system forms a semipermeable barrier between blood and the interstitium and regulates various critical biochemical functions. Collectively, it represents a prototypical biomechanical system, where the complex hierarchical architecture, from the molecular scale to the cellular and tissue level, has an intimate and intricate relationship with its biological functions. We investigated the mechanical properties of human pulmonary artery endothelial cells (ECs) using atomic force microscopy (AFM). Concurrently, the wider distribution and finer details of the cytoskeletal nano-structure were examined using fluorescence microscopy (FM) and scanning transmission electron microscopy (STEM), respectively. These correlative measurements were conducted in response to the EC barrier-disrupting agent, thrombin, and barrier-enhancing agent, sphingosine 1-phosphate (S1P). Our new findings and analysis directly link the spatio-temporal complexities of cell re-modeling and cytoskeletal mechanical properties alteration. This work provides novel insights into the biomechanical function of the endothelial barrier and suggests similar opportunities for understanding the form-function relationship in other biomechanical subsystems.

The elegance of biological systems lies in their hierarchical organization, which is programmed at the molecular scale but is manifested across various length scales with diverse functional characteristics. This very elegance of complexity and interconnected length scales and functionalities of biological systems make it exceedingly difficult to characterize the various components and their functional connectivity. Various tools and techniques have evolved during the past couple of decades that are able to address the nature and function of the isolated components, e.g., a particular protein structure or structural folding pattern or green fluorescent protein (GFP) imaging of binding or other functional characteristics of biomolecules. Because the various components occur at varied length scales and exhibit diverse characteristics, we have been developing *correlative* multiplexed microscopy/characterization as an integrative approach to address the structural complexities and functional characterization of biological systems. Our approach involves invoking correlative microscopy and characterization across appropriate length scales while simultaneously probing the functional characteristics to achieve a spatio-temporal understanding of the connectivity between the hierarchical architecture and associated cellular and tissue response.

Endothelial cells (ECs) line the vasculature and regulate various functions such as the vascular tone, blood coagulation, inflammation, angiogenesis, and tissue fluid homeostasis^1^,^2^. In the lung, ECs provide a semipermeable barrier between the vascular contents and the pulmonary interstitium/airspaces that is particularly important for the maintenance of normal fluid homeostasis and adequate gas exchange. A significant and sustained increase in vascular permeability is a hallmark of acute inflammatory diseases such as acute respiratory distress syndrome (ARDS) and is also an essential component of tumor metastasis, angiogenesis, and atherosclerosis^3^,^4^,^5^,^6^. The size-selective characteristic of the barrier to plasma proteins and other solutes is a key factor in maintaining tissue fluid balance. In addition to the biochemical functions, these processes also embody complex biomechanics. Actin filaments, which form a dynamic structural framework in the EC cytoskeleton, combine structural integrity and mechanical stability with the ability to undergo network reorganization and restructuring^7^. Agonist-induced rearrangement of actin filaments results in changes of the cell shape and altered cell-cell/cell-matrix linkage combining to modulate the EC barrier function^8^,^9^,^10^. However, the critical alterations in cell mechanics caused by the actin rearrangement as well as the effects of the altered mechanical properties on endothelial barrier permeability have yet to be fully elucidated, which is clinically important for the development of barrier-modulating therapies.

Correlations between cellular mechanical properties and various human diseases or abnormalities have recently been reported. They have been implicated in the pathogenesis of many progressive diseases, including vascular diseases^11^,^12^, cancer^13^,^14^,^15^,^16^, malaria^17^,^18^,^19^,^20^, kidney disease^21^,^22^, cataracts^23^,^24^, cardiomyopathies^25^,^26^ and Alzheimer’s dementia^27^,^28^. The alterations in the mechanical properties of cells may affect the biological and chemical responses of tissues and organs, which finally lead to various pathologies or diseases. Thus, the discovery of localized biomechanical correlations with cellular and sub-cellular architecture in terms of structural and biochemical pathways represents important issues for fundamental understanding of form-function relationships as well as development of potential therapies and intervention strategies. It is thus essential to combine disparate techniques for the same system to unravel the complex form-function relationships with adequate spatial/structural resolution and force sensitivity. In this study, the agonist-induced alteration in the local mechanical response of ECs is directly imaged and analyzed using atomic force microscopy (AFM). At the same time, we investigate cytoskeletal re-modeling and re-arrangement using fluorescence microscopy (FM) and scanning transmission electron microscopy (STEM) in response to barrier-modulating stimuli. Two well-characterized and physiologically relevant stimuli are used: thrombin, a potent barrier-disrupting agonist that causes immediate and profound EC barrier impairment, actin stress fiber formation and para-cellular gap formation^3^,^29^,^30^, and sphingosine 1-phosphate (S1P), a biologically active phospholipid generated by the hydrolysis of membrane lipids in activated platelets and other cells that produces significant EC barrier enhancement by means of peripheral actin rearrangement and ligand-receptor binding, strengthening both intracellular and cell-matrix adherence^1^,^9^,^31^,^32^. These collective and correlative results describe a functional link among the actin network organization, sub-cellular mechanical properties and endothelial barrier permeability.

## Methods and Materials

### Reagents and cell culture

All reagents [including thrombin and sphingosine-1-phosphate (S1P)] were purchased from Sigma-Aldrich unless otherwise specified. Rhodamine-phalloidin, Dulbecco’s phosphate buffered saline (D-PBS) and trypsin were purchased from Life Technologies. 16% formaldehyde used for cell fixation was from Electron Microscopy Science and bovine serum albumin (BSA) from Fisher Scientific.

Human pulmonary artery endothelial cells (HPAECs) (Lonza, Inc.) were cultured in complete growth medium consisting of Endothelial Growth Medium-2-Microvessel (EGM-2MV, Lonza) with 10% fetal bovine serum (FBS) at 37 °C in a humidified atmosphere of 5% CO_2_ and 95% air. Endothelial cells (EC) were utilized at passages 6–9 and cell culture medium was changed to EGM-2MV with 2% FBS prior to experimentation.

### AFM imaging and mechanical characterization

Cells were cultured in 60 mm diameter plastic petri dishes coated with collagen (BD Biosciences). Sub-confluent cell coverage (40–50% of petri dish area) was used to ensure the presence of single cell for AFM measurement. Before AFM imaging and measurements, cells were washed three times with sterile D-PBS to remove any debris that might stick to the AFM probe during the experiments. Growth medium was replaced with fresh medium supplemented with 2% FBS to induce a basal state in the cells prior to the experiment. AFM imaging and force measurements were performed at room temperature using a BioScope Catalyst atomic force microscope (AFM) with a Nanoscope V controller (Bruker, Inc.) sitting on the Axio Oberver.D1m (Carl Zeiss, Inc.) inverted optical microscope. The system allowed precisely laterally positioning of the silicon nitride AFM probe over the target cell.

**Figure 1:**
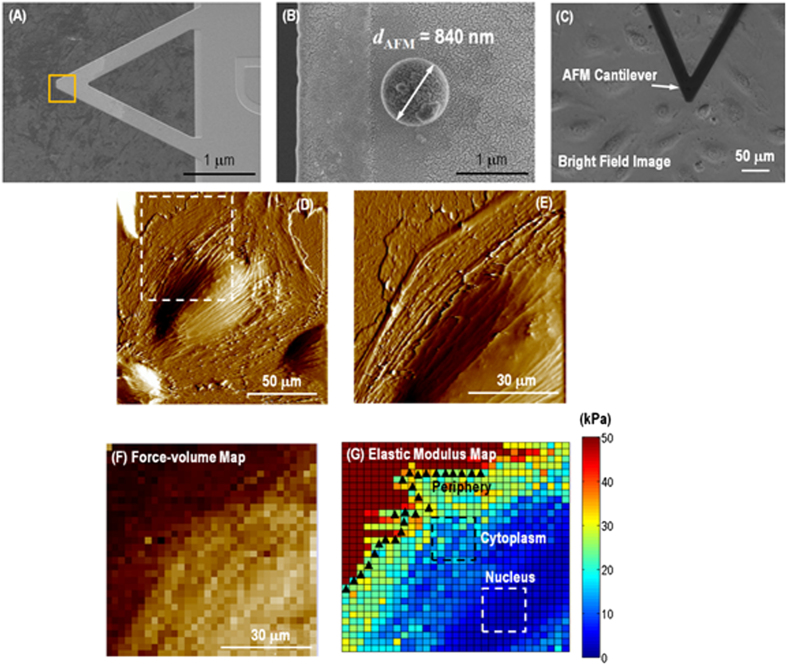
3-region analysis of force-volume map on a live single cell. (**A**) SEM image of colloidal probe with *d*_AFM_ ~ 840 nm SiO_2_ sphere attached onto a silicon nitride triangular cantilever. (**B**) Magnified SEM image of the SiO_2_ sphere. (**C**) Bright field optical image indicating colloidal AFM probe doing indentation on a single live cell. (**D**) A single spreading live cell identified by AFM scanning, showing presumably actin filament stress fibers could be visualized just under the surface of cell member and morphological details. (**E**) High resolution deflection image showing more morphological details of the regions indicated with square in (**D**), with all three nuclear, cytoplasmic and peripheral regions represented. (**F**) A force-volume image generated by the AFM indentation using colloidal probe with 32 × 32 force-displacement curves collected on 70 × 70 μm^2^ area. (**G**) Elastic modulus map generated by the force-displacement curve fitting at each pixel based on variable-indentation-depth fitting to Hertzian contact mechanics model for spherical indenter, showing soft cell compared to rigid substrate. 3 different parts were selected by isolating pixels representing the nucleus, cytoplasm and periphery regions.

Scanasyst mode in liquid was used to investigate the cells morphology alteration in response to two well-characterized and physiological stimuli (thrombin and S1P). ScanAsyst-Fluid probe (Bruker, Inc.) with a nominal spring constant *k* ~ 0.7 Nm^−1^ and a nominal tip radius *R* ~ 20 nm was employed. To reduce stress in cells during AFM mechanical measurement, SiO_2_ bead with a diameter of ~840 nm attached to a tipless silicon nitride triangular cantilever with 30 nm gold coating (Novascan Technologies, Inc.) was used for elasticity measurements as shown in Fig. 1A. The deflection sensitivity was calibrated by repeated contact mode indentation on a clean glass slide (VWR International, Inc.) in air and the spring constant of the compliant AFM cantilever was measured to be *k* = 0.12 ~ 0.15 Nm^−1^ by thermal noise method^33^. The tip radius was determined post-mortem by scanning electron microscope (SEM, Hitachi SU8030) as shown in Fig. 1B. Figure 1C is the optical microscopy image taken during the AFM probe doing indentation on the target live cell in growth medium. The loading-unloading process was conducted at roughly 1.5 μm.s^−1^ and was accomplished within roughly 1 second. The applied load, *F*, was measured a function of the vertical actuation displacement of the piezoelectric cell, *y*, throughout loading-unloading. 1024 data points were collected for each curve. A maximum load of ~2 nN was applied at each data point in order to keep the indentations on the cells within the elastic range^34^. During a typical experiment, a large scale (100 ~ 150 μm) contact mode AFM image was rapidly acquired at a resolution of 256 lines/frame to locate an individual cell appropriate for measurements as shown in Fig. 1D. A zoomed-in area was chosen containing a part of the nucleus as well as periphery and cytoplasm in order to collect localized information of mechanical properties in Fig. 1E. Measurements were carried out by acquiring arrays of 32 × 32 loading-unloading curves (force-volume map) within an area of 50 × 50 ~ 70 × 70 μm^2^ and each force-volume map was acquired over time periods of ~18 min. One force-volume map was first characterized on single EC grown on the coated petri dish before adding any stimulation. Then cells were stimulated by sequential adding solutions of thrombin and S1P to reach a final concentration of 1 unit/mL (thrombin) and 1 μM (S1P) during data acquisition. Elasticity measurements were collected in 3 time-lapse force-volume measurements on the same cell that lasted ~54 min (18 min × 3) for each stimulation. Therefore for each experiment, 7 time-lapse force-volume images were collected: 1 for unstimulated cell, 3 after thrombin stimulation and 3 after S1P stimulation. To keep the reproducibility, 3 different cells were analyzed to generate the elastic modulus time-lapse response to EC barrier-disrupting thrombin and barrier-enhancing S1P. The data collected at the cell nucleus and cytoplasm as well as in the periphery are analyzed by variable-indentation-depth fitting of force-displacement curves to spherical Hertzian contact model in order to avoid the rigid substrate effect.

According to the spherical Hertzian contact mechanical model^35^, the constitutive relation for a rigid spherical probe with radius of *R*_AFM_ pressing vertically on an elastic half continuum with elastic modulus, *E*, and Poisson’s ratio, ν = 0.50 δ_*s*_, is used to compute the cell elastic modulus, which is given by:
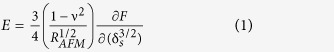


Once the elastic modulus maps as shown in Fig. 1G was generated from the force-volume maps (Fig. 1F), pixels from three different regions, i.e., nucleus, cytoplasm and periphery, were selected for localized analysis of the mechanical response. Refer to the Supplementary Information for details in data analysis.

### Fluorescence microscopy and STEM imaging

The cytoskeleton first has to be uncovered for direct electron microscopy observation and detergent lysis is the most usual way to remove the cell membrane. On the day of proceeding cell growth, EC were seeded at ~70%–80% confluence in the complete medium in a T-25 flask (Corning, Inc.). Cells were washed three times in sterile D-PBS, harvested in 0.05% trypsin, diluted into 4 mL of culture medium and centrifuged at 100 × g at 18 °C for 10 min. The supernatant were completely aspirated, cells were re-suspended in complete growth medium and then counted with a hemacytometer (Hausser Scientific, Inc.) to adjust the cell concentration to be 6 ~ 10 × 10^4^ cells/mL. Thin bar hexagonal 200 mesh gold grids with formvar/carbon coating (Electron Microscopy Science, Inc.) were purchased as substrate. Hanging droplet method was adopted for cell growth on the TEM grid^36^. The grid with formvar facing up was plasma cleaned with Argon gas for 20 seconds at 20 Watts in advance. A 50 μL droplet of the cell suspensions was pipetted onto the inside of the cover lid of a sterile plastic petri dish with a diameter of 35 mm (Corning, Inc.) and the lid was flipped over so that the droplet was hanging upside down. With the formvar side facing the droplet, a TEM grid was placed on the droplet as a substrate for cell growth. This cover lid was then placed on the base of the petri dish and put inside the incubator for cells attachment. After 1 hour, the TEM grids were transferred from the hanging droplets onto 3 mL warm complete growth medium in a fresh 35 mm petri dish and attached cells were allowed to grow for another 10 hours inside the incubator. 3 grids were prepared simultaneously from the same batch and passage of cells to test the effects of thrombin and S1P on the re-modeling of endothelial cytoskeleton nano-structure. Before treatment with any stimulation, the grid handled with a home-made Pt-wire loop was briefly rinsed three times on PBS droplets on the surface of a parafilm and floated on 1 mL growth medium supplemented with 2% FBS with cell facing the liquid in a sterile 35 mm petri dish. One grid acted as a control sample, the second was treated with thrombin (1 unit/mL) for 18 min, and the last one with S1P (1 μM) for 18 min, to correspond to the time needed for collecting each frame of AFM force-volume image. The cells were incubated inside the incubator during the stimulation. Before sample treatment, the sample was briefly rinsed three times with PBS again and cells were immediately extracted with 150 μL extraction buffer composed of 1% non-ionic detergent Triton X-100 and 4% PEG (MW 40,000) in buffer M for 15 min with 3 changes, followed by stabilization in 150 μL M buffer (50 mM imidazole, 50 mM KCl, 0.5 mM MgCl_2_, and 0.1 mM EDTA with final pH adjusted to 7.1 using concentrated HCl) for 15 min with 3 changes. The cells were then fixed with 150 μL of 4% formaldehyde for 15 min and then washed three times with PBS. Visualization of fluorescently stained actin filaments was achieved with the following method: Following fixation, unreacted aldehyde groups were quenched with 0.05 M glycine for 15 min. Non-specific binding sites were blocked with blocking buffer (0.25% fish skin gelatin, 0.01% saponin, 0.1% NaN_3_ in PBS) for 15 min with 3 changes, 5% bovine serum albumin (BSA) in blocking buffer for 15 min as 2^nd^ step blocking. Fluorescent phalloidin was adopted to extensively use as a probe for actin filament observation under fluorescence microscopy^37^. Actin filaments were labeled with ~0.165 mM rhodamine-phalloidin for 30 min and with 0.5 μg/mL DAPI as a counterstain for nucleus identification for 5 min at room temperature with protection from ambient light. After labeling with rhodamine-phalloidin and DAPI, the grids were briefly rinsed three times with Millipore water droplets, placed inside a glass bottom petri dish with 2 mL Millipore water and analyzed using an Axio Oberver. D1m fluorescence light microscope (Carl Zeiss, Inc.) with an AxioCam MRc CCD camera. After observation under fluorescence microscopy, the TEM grids were put into Millipore water-filled porous capsule (Electron Microscopy Science, Inc.) and dehydrated through a series of ascending ethanol concentrations of 10%, 25%, 50%, 75%, 90%, and 100% for 5 min each, followed by critical point drying in a Samdri-795 critical point dryer (Tousimis Inc.). Finally, actin filament fine nano-structure was captured using scanning transmission electron microscope (STEM, Hitachi HD 2300) with an accelerating voltage of 80 kV. The digital images obtained from both microscopes were transferred to Adobe Photoshop for color adjustment and figure overlay.

## Results and Analysis

### Alteration of cell morphology in response to barrier-regulatory stimuli

**Figure 2:**
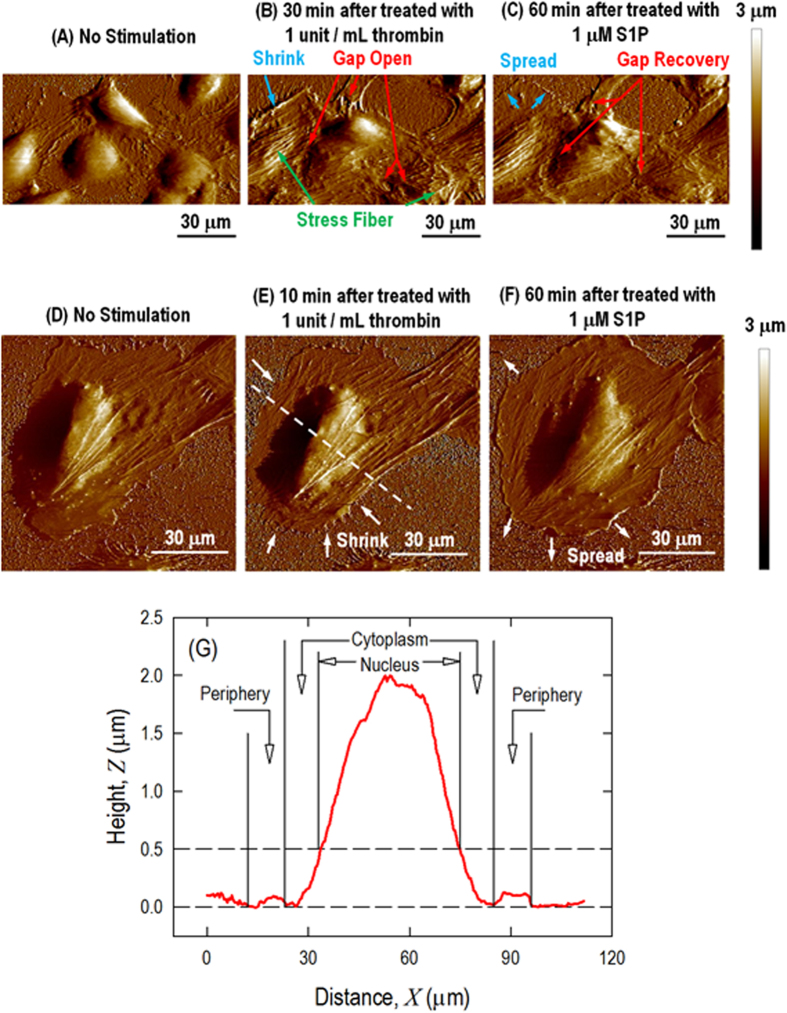
AFM scanning images of HPAEC monolayer (**A**, **B**, **C**) and a single (**D**, **E**, **F**) live cell in response to thrombin and S1P. Monolayer pulmonary EC grown on collagen-coated plastic petri dish with (**A**) no stimulation, exhibiting a polygonal shape with a cobblestone appearance without any apparent intercellular gap; (**B**) thrombin (1 unit/mL, 30 min), illustrating that central filament bundles begin to appear and become aligned with the long axis of the cells and that intercellular gaps begin to appear locally due to cell lateral contraction; and (**C**) S1P (1 μM, 60 min), illustrating the formation of the cortical actin ring and recovery of the intercellular gap. A single pulmonary EC grown on a plastic petri dish with (**D**) no stimulation, illustrating that the cell is well spread on the substrate and that actin filaments are distributed along the edge of the cells, forming peripheral bundles; (**E**) thrombin (1 unit/mL, 10 min), illustrating the thrombin-induced cell retraction response; and (**F**) S1P (1 μM, 60 min), inducing the respreading of the cell periphery on the substrate and the formation of the cortical actin ring along the cell periphery. (**G**) Cross-section profiles of live cells showing the selection of 3 different regions of the cell labeled as the nucleus, cytoplasm and periphery.

Under a physiologically viable fluidic specimen stage, live ECs are analyzed using serial high resolution AFM imaging (256 lines/frame) after the sequential addition of thrombin to model barrier disruption, followed by S1P to stimulate barrier recovery. The concentration is selected to mimic the natural physiological processes occurring during lung injury events in human blood vessels for thrombin (1 unit/mL), while 1 μM S1P has been observed to produce rapid and dramatic enhancement of polymerized F-actin and myosin light chain phosphorylation at the cell periphery^1^,^38^. The endothelial barrier function is highly dependent on a complex balance between the intracellular contractile forces generated by actin-myosin contraction and the cellular adhesive/resistive forces produced by cell-cell/cell-matrix interactions, and rigid cytoskeletal components (microtubules and intermediate filaments)^2^,^39^. Figure 2 shows the monolayer and a single live EC morphology alteration in response to thrombin and S1P by ScanAsyst mode scanning in liquid. The monolayer pulmonary ECs grown on collagen-coated plastic petri dishes have a polygonal shape in cobble-stone appearance with an intact border and no apparent intercellular gaps as observed in Fig. 2A. After incubation with thrombin for 30 min, long and thick stress fibers begin to appear and become aligned with the long axis of the cells (the green arrows in Fig. 2B) associated with the disappearance of lamellipodia. Because the cell-cell/cell-matrix adhesion cannot balance the contractile forces induced by the thick transverse stress fibers inside individual cells, multiple large gaps are observed between adjacent cells (red arrows). The increased cytoskeletal density in the nuclear and cytoplasmic regions causes a loss of focal adhesion and finally causes the entire cell to shrink on the substrate (blue arrow). The previously observed thick stress fibers are reduced 60 min after the addition of S1P. The cortical actin ring is formed along the cell periphery and induces the cell to respread out on the substrate, as observed in Fig. 2C (blue arrow), leading the intercellular gaps start to recover (red arrow).

The changes in the endothelial barrier function in a confluent monolayer within blood vessels are a consequence of integrated changes in the cytoskeletal nano-structure at the level of each individual cell. However, examining the changes in cell monolayers *in vitro* is complicated because of complex cell-cell interactions. Therefore, we instead analyze individual cell as a simplified system to advance our understanding of the correlation between the mechanical properties and the cytoskeletal nano-structure arrangement.

Without cell-cell interactions, the thrombin and S1P effects are more prominent and faster on the single-cell level. Fig. 2D shows the characteristics of an individual pulmonary EC. The EC is well spread on the substrate, and actin filaments are distributed along the edge of the cells, forming peripheral bundles. After incubation with thrombin for only 10 min, thick fibers are induced (arrows, Fig. 2E) that round up the cell leading to cell retraction, which is the primary mechanism for intercellular gaps forming in the monolayer. S1P (1 μM, 60 min) stimulates the respreading of the cell periphery on the substrate and the formation of the cortical actin ring along the cell periphery, as observed in Fig. 2F, which stabilizes cell-cell junctions and is associated with the recovery of intercellular gaps. The cross-section profile is shown in Fig. 2G with the selection of 3 different regions labeled as the nucleus, cytoplasm and periphery. The following measurements represent the baseline heights of the pulmonary ECs based on measurements of 10 live cells: nuclear region 1.57 ± 0.31 μm; cytoplasmic region 0.39 ± 0.11 μm; and peripheral region 0.15 ± 0.09 μm.

### AFM mechanical characterization

**Figure 3:**
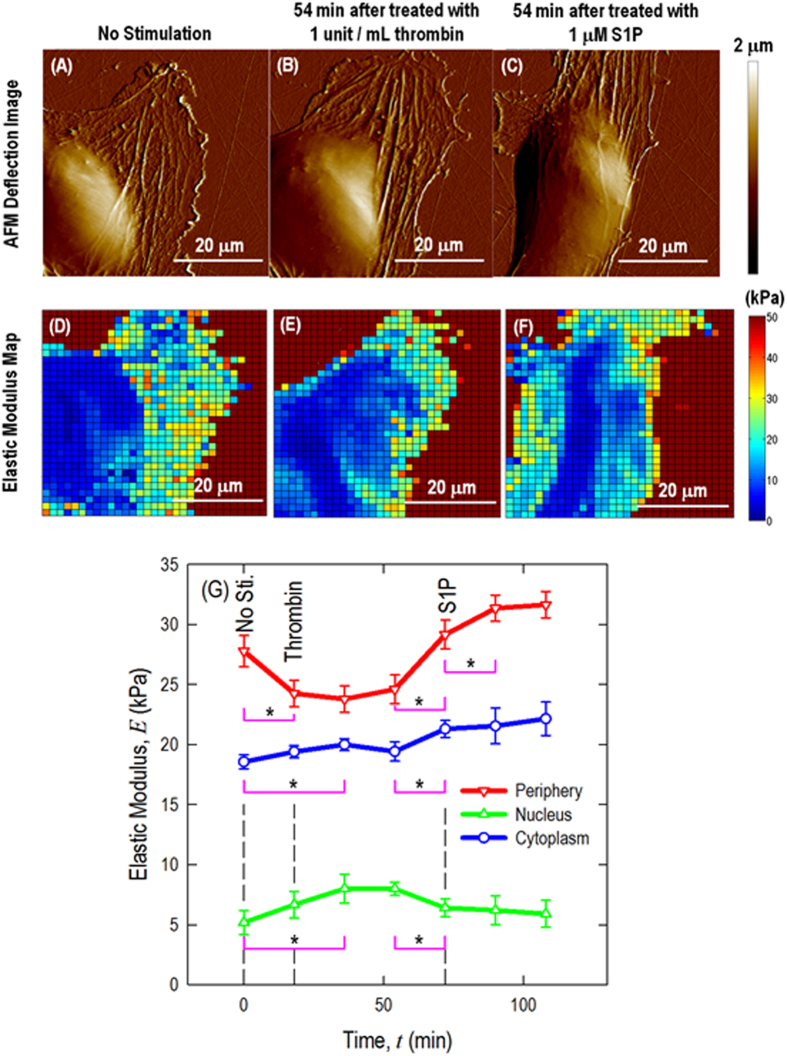
AFM deflection images and time-lapse elastic modulus maps of live EC in response to (**A**, **D**) no simulation, (**B**, **E**) barrier-disrupting thrombin (1 unit/mL, 54 min) and (C, F) barrier-enhancing S1P (1 μM, 54 min); (**G**) quantification of live cell sub-cellular elastic modulus as a function of time in response to sequential thrombin (1 unit/mL) and S1P (1 μM) treatment. *n* = 3 different cells are analyzed to generate the elastic modulus time-lapse responses. *p < 0.05.

When probed, the elastic resistance to AFM indentation reflects the rigidity of the actin network and the actin stress fiber stiffness arises from increased actomyosin interaction and crosslinking of long, thick cables of actin. Actin provides mechanical reinforcement for the cytoplasm. To study the effects of cytoskeletal organization on the mechanical properties of live pulmonary ECs, AFM measurements on an individual cell are performed before and after stimulation with the barrier-modulating agents, thrombin and S1P. The data collected at the cell nucleus and cytoplasm as well as in the periphery are analyzed to correlate these biomechanical properties with detailed information on the cytoskeletal actin nano-structure from the fluorescence microscopy and STEM studies described below. Figure 3 presents the AFM deflection images and time-lapse elastic modulus maps of a live EC in response to (A, D) no simulation, (B, E) barrier-disrupting thrombin (1 unit/mL, 54 min) and (C, F) barrier-enhancing S1P (1 μM, 54 min).

The cellular mechanical property is characterized using contact mode AFM scanning with a colloidal probe (*d*_AFM_  ~ 840 nm, as shown in Fig. 1B) and a resolution of 256 lines/frame. Force-volume mapping, with a resolution of 32 lines/frame, is performed after switching to force-volume mode, and elastic modulus maps are generated by curve fitting to the spherical Hertzian contact model demonstrated in the Supplementary and Figure S1. In addition to the thrombin-induced cell retraction and S1P-induced cell re-spreading described above (Figs 3A–C), more quantitative information could also be obtained from these mechanical characterizations (Figs 3D–F). The live EC sub-cellular elastic modulus responses are plotted in Fig. 3G. For the baseline, the highest elastic modulus is observed at the cell periphery, followed by in the cytoplasm and cell nucleus. Stimulation of the EC with thrombin induces rapid transversal stress fiber formation in the nuclear and cytoplasmic regions as well as cellular retraction on the substrate. The peripheral elastic modulus decreases significantly (*p < 0.05, determined by a two-tailed unpaired Student’s *t*-test), while the nuclear and cytoplasmic regions slightly rise over time. This pattern correlates with a decrease in the peripheral cortical actin nano-structure and the assembly of cytoskeletal stress fibers in the central part, which yields a contractile phenotype associated with intercellular gap formation.

The mechanical properties begin to change in the first frame of the AFM mechanical characterization after thrombin treatment (~18 min) and are prominent after ~36 min of treatment. After the addition of S1P, the peripheral elastic modulus increases significantly (*p < 0.05), which correlates with peripheral cortical actin that stabilizes cell-cell junctions, reverses the thrombin effect, and results in closing of intercellular gaps when the cells are in a monolayer. Prior work has demonstrated that maximal barrier enhancement is observed with the use of 1 μM S1P, peaks after 30–40 min and is sustained during the entire testing period based on pharmaceutical study^1^. Figure S2 presents the entire time series cell elasticity maps in response to barrier-disrupting thrombin and barrier-enhancing S1P. Our results indicate that the elastic moduli of the peripheral and nuclear regions change significantly in response to the barrier-altering stimuli and theses alterations correlate with the known changes in the EC permeability.

### Fluorescence microscopy and STEM imaging

To obtain fundamental and qualitative structural information about actin filaments with different resolutions, correlative multiplexed microscopes with various resolution limits are used to precisely analyze the same nano-structures within a single cell using both fluorescence microscopy (FM) to examine a wider distribution of filaments at the cellular level and scanning transmission electron microscopy (STEM) to examine the finer structural details with molecular level resolution. This correlative imaging allows additional novel morphological information about the actin filament to be obtained, which provides a degree of confidence about the nano-structure of interest, as information obtained using one method can be compared with that obtained using other methods.

**Figure 4:**
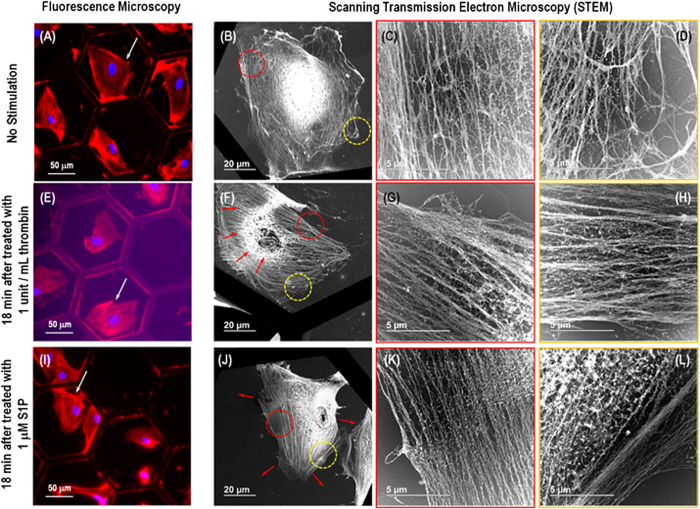
Correlative fluorescence microscopy and STEM images showing the same nano-structures within a single pulmonary EC for both a wider distribution and finer structural details of filaments. Images (**A**-**D**), (**E**-**H**) and (**I**-**L**) correspond to unstimulated, thrombin-treated (1 unit/mL, 18 min) and S1P-treated (1 μM, 18 min) cells, respectively. (**A**), (**E**) and (**I**) present florescence microscopy images of unstimulated, thrombin-treated and S1P-treated cells, respectively. (**B**), (**F**) and (**J**) are STEM images of the entire unstimulated, thrombin-treated and S1P-treated cell pointed by the white arrows in (**A**), (**E**) and (**I**), respectively. (**C**-**D**), (**G**-**H**) and (**K**-**L**) are zoomed-in STEM images revealing the fine nano-structures of the actin filament in the areas encircled with red and yellow dotted lines in (**B**), (**F**), and (**J**), respectively. The red arrow in (**F**) indicates the cell after treated by thrombin (1 unit/mL, 18 min) is contracted in shape and that in (**J**) shows dense cortical actin band resulting from S1P treatment (1 μM, 18 min).

Figure 4 presents the correlative fluorescence microscopy and STEM images of precisely the same nano-structures within a single pulmonary EC. In the fluorescence microscopy images, the red color represents the rhodamine-phalloidin staining of actin filaments, and the blue represents cell nuclei. For the EC without any stimulation, cobblestone morphology is observed with actin filaments being primarily distributed at the cell periphery (Fig. 4A). Figs 4B–D present STEM images of the entire cell as well as the cytoplasm/periphery region. These images demonstrate that the cell is well spread on the TEM grid, the cytoplasm contains active fibers with random distribution and few stress fibers, and the cell periphery has a relatively low density of fibers distributed in all directions. In contrast, the FM image of the thrombin-stimulated (1 unit/mL) cell in Fig. 4E reveals robust stress fiber formation. The cell is contracted in shape (the red arrows in Fig. 4F), and the cell periphery and nuclear regions contain thick stress fibers, as observed in the STEM images of Figs 4F–H. Most of these fibers in each region appear aligned and oriented together as thick stress fibers. The FM image of the pulmonary EC after S1P treatment (Fig. 4I) reveals membrane protrusions with a high concentration of actin at the cell periphery. The STEM images reveal cell spreading with a reduction of the thick stress fibers observed after thrombin treatment. The cytoplasmic region again consists mostly of thin actin fibers with random arrangement. The cell peripheral region reveals the formation of a dense cortical actin band resulting from S1P treatment, as observed in Figs 4J–L. This dense band correlates with an increase in the elastic modulus of the cell peripheral regions after exposure to S1P, as revealed by AFM. In addition, the nuclear region also shows the disappearance of stress fibers after S1P treatment. This correlation in the fiber nano-structure observed in STEM and fluorescence images is a strong indicator that cell remodeling observations are affected by actin fiber rearrangements and these rearrangements result in cellular stiffness redistribution from the central nucleus and cytoplasm to the periphery as shown from AFM mechanical characterization. Figure S3 presents an STEM image of single actin filament, demonstrating that the diameter is ~7 nm.

## Discussion

The vascular endothelium is both a cellular target and key participant in the profound physiologic derangement that accompanies inflammatory lung injury with vascular hyper-permeability and subsequent barrier restoration serving as key expressions of this involvement. Changes in cellular mechanical properties are important pathophysiologic steps in multiple disease processes. Thus, the discovery of localized biomechanical property correlations with the sub-cellular nano-structure represents an important issue for both fundamental structural-function relationships and potential future therapy development.

Thrombin is a proteolytic enzyme that forms from prothrombin and plays a critical role in the blood coagulation process. It produces increased levels of Ca^2+^, which activates myosin on actin filaments to produce stress fibers in the central region of the cell and hence induces cell contraction in ECs^40^. In contrast, S1P, a phospholipid generated by the hydrolysis of membrane lipids in activated platelets and other cells, increases the level of the actin-polymerizing protein, cortactin and the myosin activity at the cell edges, increasing cell tethering forces and cell spreading to enhance the EC barrier function^41^,^42^. Thus, thrombin and S1P induce completely opposite of the actin filament remodeling.

In the current study, we evaluate the cell mechanical properties in response to well-characterized and physiologically relevant barrier-regulatory stimuli using dynamic live cell AFM force-volume mapping. Concurrently, the cytoskeletal structural re-arrangement is evaluated using fluorescence microscopy and scanning transmission electron microscopy. Localized AFM mechanical measurements and actin filament nano-structures observation from fluorescence microscopy/STEM indicate that live cells respond to thrombin and S1P in accordance with the proposed barrier-modulation model. Actin filament stress fibers could be visualized just under the surface of cell member in AFM images and observed directly in STEM and fluorescent optical microscopy images. For the untreated cells as shown in Fig. 1D, Fig. 2A, D, Fig. 3A and Fig. 4A–D, fine actin fibers are transverse the cell. However, both thrombin and S1P serve to initiate dynamic cytoskeletal rearrangement of actomyosin fibers with dramatic agonist-specific alterations in the intracellular distribution. Thrombin induces cell contraction and disappearance of lamellipodia associated with newly formed thick, prominent cytoplasmic actin stress fibers as shown in Fig. 2B, E, Fig. 3B and Fig. 4E–H. This causes increased elastic modulus in the central region of the cell and decreased stiffness in the periphery. In contrast, S1P reverses the effect of thrombin and induces formation of dense cortical actin rings to stabilize EC and the active ruffling lamellipodia as shown in Fig. 2C, F, Fig. 3C and Fig. 4I–L. This process decreases the stiffness at the cell nucleus and increases the elastic modulus at the periphery. These stress fibers play a major role in the deformation resistance of endothelial cells, which are consistent with our previous work using fixed pulmonary ECs^43^ and live human lung microvascular EC^44^, demonstrating the robust and reproducible nature of these biomechanical correlations. These observations in the AFM mechanical properties measurements are correlated with fluorescence microscopy and STEM imaging results to provide novel insights concerning the mechanisms by which thrombin induces thick actin stress fiber bundles formation transverse the cells and S1P increases the formation of peripheral actin nano-structures. There are several other different barrier-disruptive agonists such as VEGF, H_2_O_2_ and barrier-enhancing agonists such as OxPAPC, HGF and prostacyclin, which can induce similar phenomena as those treated with thrombin and S1P as shown in the manuscript. Rac GTPase, plays critical roles in the peripheral actin cytoskeletal remodeling and cell-cell junction assembly, which is activated in ECs stimulated with OxPAPC, HGF, and prostacyclin. In contract, activation of Rho GTPase and MLCK, which promote central stress fiber formation, cell retraction, and disruption of cell-cell contacts, has been described in ECs challenged with thrombin, H_2_O_2_ and VEGF.

The changes in the mechanical properties of cells may affect the biological and chemical responses of tissues and organs, which finally lead to various pathologies or diseases. However, conventional pharmaceutical and biological research uses the biochemical approach, while the obviously related biomechanical aspects are largely ignored. Our studies provide new insights into the regulation of biomechanical properties in ECs using barrier-modulating agents and demonstrate the strong correlation of these biomechanical properties with the cellular/sub-cellular nano-structure, both spatially and temporally, using a correlative multiplexed microscopy method. This method provides information to help understand how the EC barrier functions from biomechanical perspective. The studies reported herein offer additional support for the development of clinical and pharmaceutical approaches to understand the basic mechanism of endothelial cell barrier remodeling. Moreover, this work suggests that similar opportunities may exist to understand the form-function relationship in other biomechanical subsystems.

## Additional Information

**How to cite this article**: Wang, X. *et al.* Nano-Biomechanical Study of Spatio-Temporal Cytoskeleton Rearrangements that Determine Subcellular Mechanical Properties and Endothelial Permeability. *Sci. Rep.* **5**, 11097; doi: 10.1038/srep11097 (2015).

## Supplementary Material

Supplementary Information
