# Supplementary material for: Nano-Biomechanical Study of Spatio-Temporal Cytoskeleton Rearrangements that Determine Subcellular Mechanical Properties and Endothelial Permeability
Source: Sci Rep. 2015 Jun 18;5:11097. doi: 10.1038/srep11097 (PMC4650616; doi:10.1038/srep11097)
Supplement: Supplementary Information [file srep11097-s1.pdf]

Supplementary Material for

Nano-Biomechanical Study of Spatio-Temporal Cytoskeleton Rearrangements for Subcellular Mechanical Properties-Endothelial Barrier Permeability Relationship

Xin Wang[1], Reiner Bleher[1], Mary E. Brown[2], Joe G. N. Garcia[3], Steven M. Dudek[2], Gajendra S. Shekhawat[1], Vinayak P. Dravid[1*]

Supplementary Materials:

Methods and materials

<u>Data analysis</u>

The AFM cantilever with spring constant of k, connected to the piezoelectric cell, is used to deform the cell while the interaction force, F, is detected based on the deflection of the cantilever, δ_c. The raw AFM data is the relationship between force, F, and piezoelectric cell displacement, y, where $F = k \times \delta_c$. Since the AFM cantilever is compliant, it is bending into the opposite direction, δ_c, while the sample is indented by δ_s. Thus displacement traveled by the piezoelectric cell, y, does not correspond to the actual tip distance. Raw AFM force-displacement is the relationship between (F vs. y) needs to be converted to the relation between applied force and cell deformation (F vs. δ_s) by correcting cantilever deflection from the piezo displacement. Upon intimate contact of the tip with the cell surface, δ_s is calculated by subtracting the cantilever deflection, δ_c, from the measured piezoelectric cell displacement, y. According to the spherical Hertzian contact mechanical model (3), the constitutive relation for a rigid spherical probe with radius of R_{AFM} pressing vertically on an elastic half continuum with elastic modulus, E, and Poisson's ratio, $\nu = 0.50$, is used to compute the cell elastic modulus, which is given by

$$E = \frac{3}{4}\left(\frac{1-\nu^2}{R_{AFM}^{1/2}}\right)\frac{\partial F}{\partial(\delta_s^{3/2})} \qquad \text{(Eqn 1)}$$

A home-made MATLAB (MathWorks, Inc.) code was used to obtain elasticity maps by analyzing all 1024 force curves in each 32 × 32 pixel force-volume image, where the goodness values R^2 exceeded 0.85 in all curve fittings. Elastic property at each

pixel was characterized by variable-indentation-depth fitting of force-displacement curve to spherical Hertzian contact model. The indentation depth at each pixel was controlled within 10% of cell thickness based on local cell thickness from AFM height image in order to minimize rigid substrate effect. A line of zero force was defined from the average deflection of points in the force-displacement curve corresponding to the positions of the cantilever when it was far away from the surface. Figure S1B is the representative raw AFM force-displacement curves collected at cell nucleus, cytoplasm, and periphery as well as rigid substrate as shown in Figure S1A and Figure S1C is the curve fitting to spherical Hertzian mechanical model for elastic modulus in the log-log plot. Figure S2 is the time-lapse elastic modulus maps of EC from AFM mechanical characterization at different time in response to thrombin and S1P. Figure S3 is the STEM image of single actin filament fiber with diameter ~ 7 nm. Table 1 is the elastic modulus of nucleus, cytoplasm and periphery regions of EC in response to thrombin and S1P.

References

1. Hutter, J.L. and Bechhoefer, J., *Calibration of Atomic-Force Microscope Tips (Vol 64, Pg 1868, 1993).* Review of Scientific Instruments, 1993. **64**(11): p. 3342-3342.
2. Li, Q.S., Lee, G.Y.H., Ong, C.N., and Lim, C.T., *AFM indentation study of breast cancer cells.* Biochemical and Biophysical Research Communications, 2008. **374**(4): p. 609-613.
3. Sneddon, I.N., *The relation between load and penetration in the axisymmetric boussinesq problem for a punch of arbitrary profile.* International Journal of Engineering Science, 1965. **3**(1): p. 47-57.
4. Svitkina, T.M., Verkhovsky, A.B., and Borisy, G.G., *Electron microscopy of the cytoskeleton of cultured cells.* Recent Advances in Microscopy of Cells Tissues and Organs, 1997: p. 93-100.
5. Quaedackers, J.A., van Engeland, S., Mutsaers, P.H.A., de Goeij, J.J.M., Snoeckx, L.H.E.H., de Voigt, M.J.A., and van der Vusse, G.J., *Determination of the intracellular element concentrations in cultured H9c2 cells by proton microprobe techniques.* Nuclear Instruments & Methods in Physics Research Section B-Beam Interactions with Materials and Atoms, 1999. **158**(1-4): p. 405-411.
6. Wulf, E., Deboben, A., Bautz, F.A., Faulstich, H., and Wieland, T., *Fluorescent Phallotoxin, a Tool for the Visualization of Cellular Actin.* Proceedings of the National Academy of Sciences of the United States of America, 1979. **76**(9): p. 4498-4502.

Supplementary Figure Captions

Fig. S1. Curve fitting to spherical Hertzian mechanical contact model at different regions of the cell as well as on the rigid substrate for elastic modulus characterization. (A) Three

different regions selected from one single cell as cell nucleus, cytoplasm, and periphery as well as rigid substrate. (B) The representative raw loading curves collected at the different regions of cells. (C) The curve fittings to spherical Hertzian mechanical model for elastic modulus in the log-log plot.

Fig. S2. Time-lapse elastic modulus maps of live EC from AFM mechanical characterization in response to barrier-disrupting thrombin (1 unit/mL) and barrier-enhancing S1P (1 μM).

Fig. S3. STEM image of single actin filament fiber with the diameter ~ 7 nm.

Table Captions:

Table 1. Time-lapse elastic modulus of nucleus, cytoplasm and periphery regions of ECs in response to 2 different stimuli: thrombin (1 unit/mL) and S1P (1 μM).

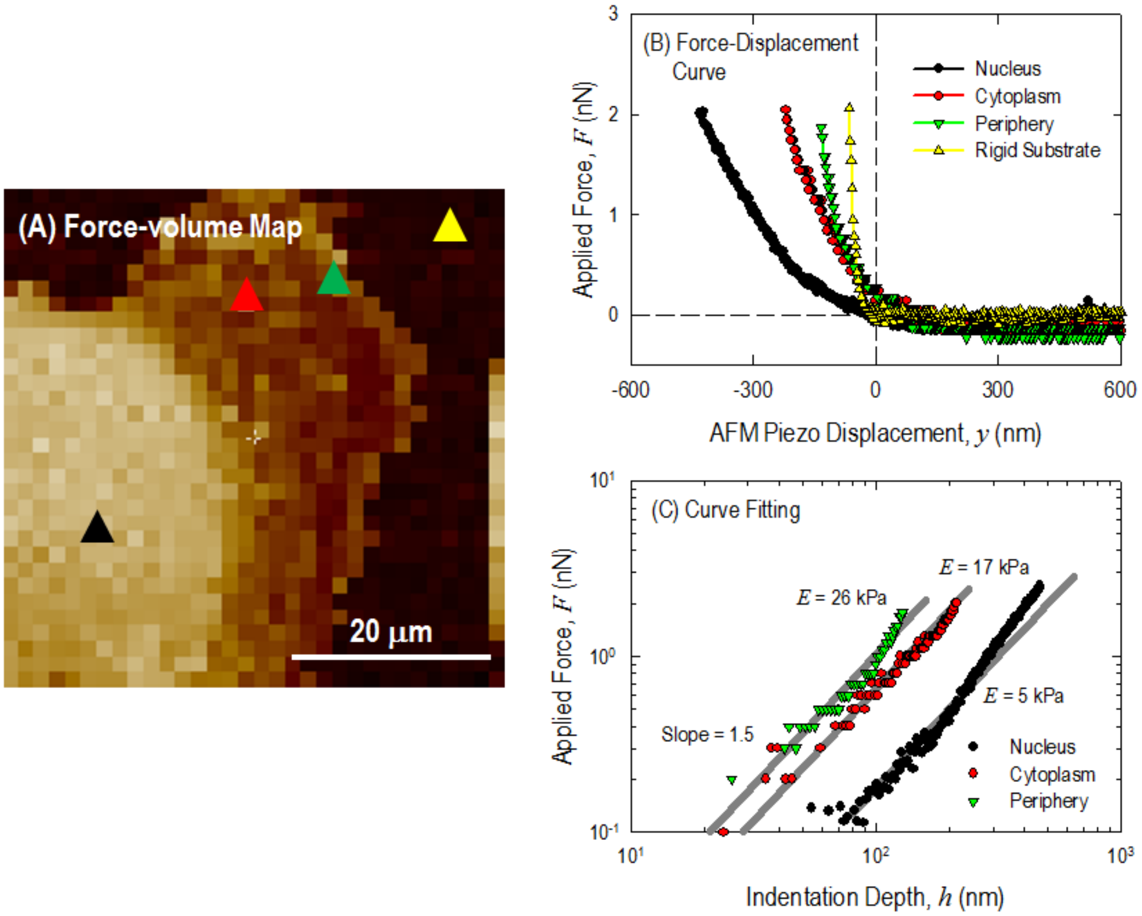

(A) Force-volume Map
20 μm
(B) Force-Displacement Curve
Nucleus
Cytoplasm
Periphery
Rigid Substrate
Applied Force, F (nN)
AFM Piezo Displacement, y (nm)
3
2
1
0
-600
-300
0
300
600
(C) Curve Fitting
E = 26 kPa
E = 17 kPa
E = 5 kPa
Slope = 1.5
Nucleus
Cytoplasm
Periphery
Applied Force, F (nN)
Indentation Depth, h (nm)

Figure S1

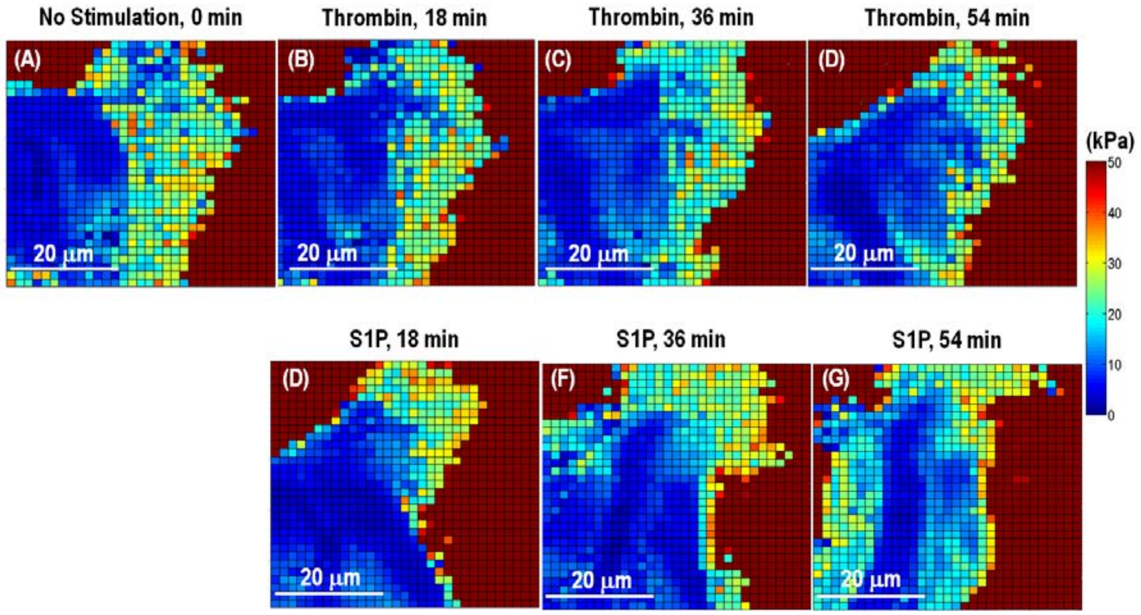
No Stimulation, 0 min
Thrombin, 18 min
Thrombin, 36 min
Thrombin, 54 min
(A)
(B)
(C)
(D)
20 μm
(kPa)
50
40
30
20
10
0
S1P, 18 min
S1P, 36 min
S1P, 54 min
(D)
(F)
(G)

Figure S2

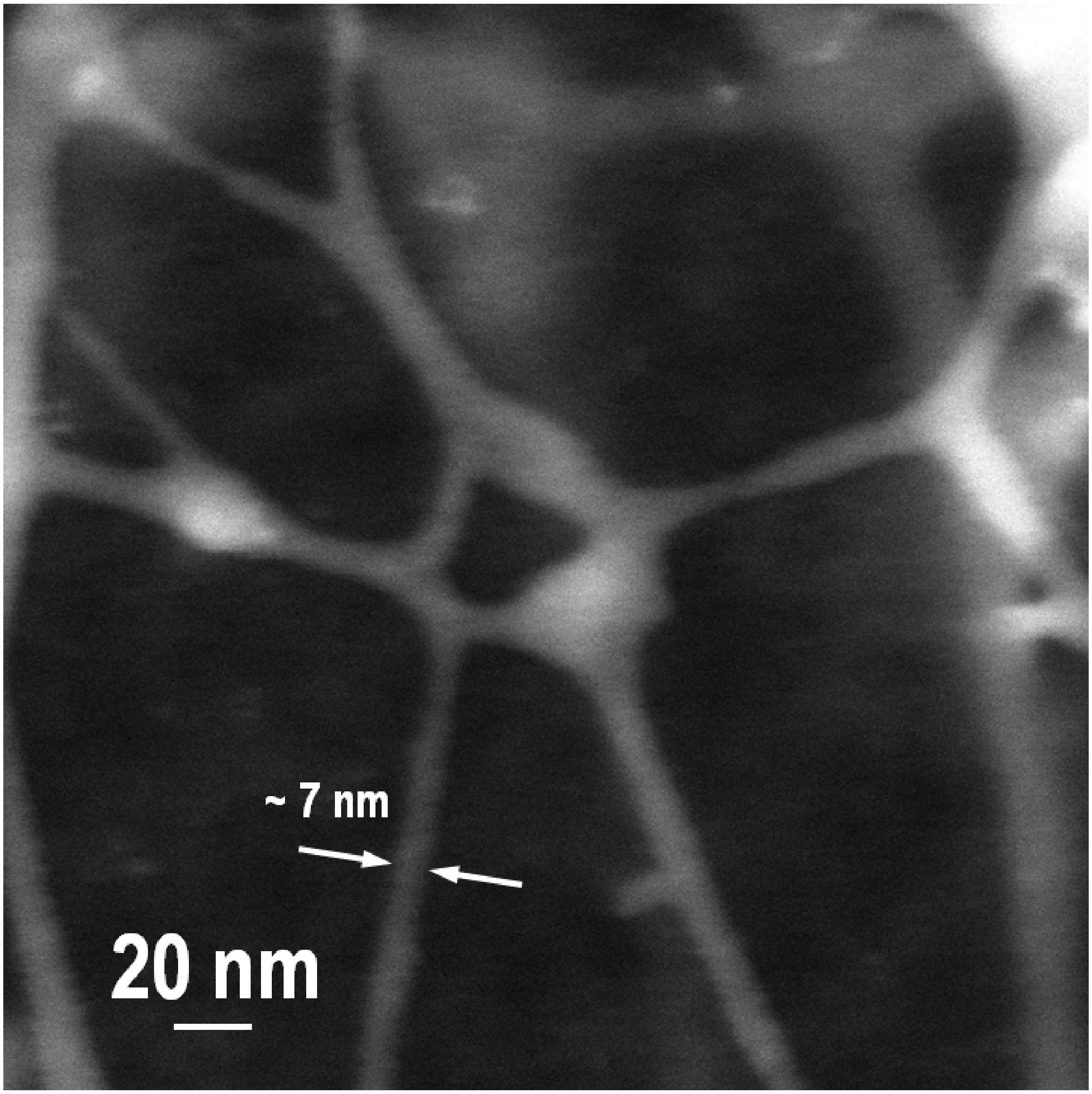

Figure S3

	Time, t (min)	Elastic Modulus of Nucleus, E_1 (kPa)	Elastic Modulus of Cytoplasm, E_2 (kPa)	Elastic Modulus of Periphery, E_3 (kPa)
No Stimulation	0	5.18 ± 1.0	18.56 ± 0.6	27.78 ± 1.3
Thrombin (1 unit/mL)	18	6.67 ± 1.09	19.40 ± 0.5	24.26 ± 1.1
	36	8.0 ± 1.2	20.0 ± 0.5	23.79 ± 1.1
	54	7.99 ± 0.55	19.42 ± 0.8	24.60 ± 1.2
S1P (1 μM)	72	6.4 ± 0.75	21.3 ± 0.7	29.15 ± 1.2
	90	6.2 ± 1.2	21.55 ± 1.5	31.35 ± 1.1
	108	5.9 ± 1.13	22.15 ± 1.4	31.64 ± 1.1

Table 1
